# Video-Based Health Promotion for Rural and Underserved Populations: A Narrative Review With Implications for Rural Alabama

**DOI:** 10.7759/cureus.117765

**Published:** 2026-10-11

**Authors:** John McCarus, Mahathi Mula, Amanda Geary, Joseph Budisak, Skylar E Nollenberger, Faraz Ahmed, Ruby Jewell, Shane Warren

**Affiliations:** 1 Medical School, Alabama College of Osteopathic Medicine, Dothan, USA; 2 Research, Alabama College of Osteopathic Medicine, Dothan, USA

**Keywords:** digital health, health literacy, health promotion, rural health, video-based education

## Abstract

Rural populations in the United States experience persistent health disparities associated with limited healthcare access, socioeconomic constraints, transportation barriers, healthcare workforce shortages, and reduced availability of health education resources. These challenges are particularly relevant in rural Alabama, where a substantial proportion of the population resides in rural communities and access to healthcare services varies considerably across geographic regions. Digital health promotion media, particularly video-based interventions, represent a potentially scalable and cost-efficient approach to supplementing traditional health education. This narrative review examines the effectiveness and potential applications of health promotion videos in improving health literacy, health behaviors, preventive healthcare utilization, and health-related outcomes among rural and underserved populations, with particular emphasis on applicability to rural Alabama. The reviewed literature includes randomized controlled trials, systematic reviews, qualitative studies, and epidemiologic sources examining video-based health education delivered through social media, online platforms, e-learning programs, and physical media. Overall, the literature suggests that video-based health education can improve health knowledge and literacy and may support medication adherence, preventive screening, healthy lifestyle practices, and chronic disease self-management. However, effects on sustained behavior change are less consistent, and outcomes vary according to intervention design, population characteristics, delivery platform, and duration of follow-up. Barriers including digital access, health literacy, misinformation, and differences in technology use must also be considered. Health promotion videos therefore represent a promising complementary strategy for addressing health education barriers in rural and underserved populations, although additional research is needed to determine how best to implement and evaluate these interventions in rural U.S. communities.

## Introduction and background

Rural communities in the United States experience substantial disparities in healthcare access and health outcomes. Individuals living in rural areas may encounter geographic isolation, transportation limitations, healthcare workforce shortages, and reduced availability of healthcare facilities compared with residents of urban areas [1-3]. These challenges can make it difficult for rural residents to obtain preventive services, chronic disease management, and timely medical care. Healthcare infrastructure also varies considerably across rural regions, with some communities experiencing limited access to hospitals, primary care providers, and other healthcare resources [1-2].

These barriers extend beyond the availability of medical treatment. Access to health education is also an important component of population health. Individuals must be able to obtain, understand, evaluate, and apply information regarding disease prevention, medication use, nutrition, physical activity, screening, and appropriate health care utilization. When healthcare professionals and education resources are geographically limited, rural residents may have fewer opportunities to receive individualized health education. Consequently, interventions that can deliver reliable health information outside traditional clinical settings may provide an important supplement to rural healthcare services.

These challenges are particularly relevant in Alabama. Approximately 42% of Alabama residents live in rural areas, and rural communities throughout the state experience differences in health care availability and infrastructure [3]. Geographic data also identify medically underserved areas and variation in the distribution of healthcare resources throughout the state [4,5]. Community health centers and related facilities provide important services in many underserved communities, but access to these resources remains geographically variable [6]. Alabama also has a substantial chronic disease burden. According to the Centers for Disease Control and Prevention, 13.9% of Alabama adults had diagnosed diabetes in 2022 [7]. The combination of chronic disease burden and limitations in health care access highlights the importance of interventions that can provide health education beyond traditional clinical encounters.

Digital technology has created new opportunities to expand the reach of health education. Health promotion videos can present information using a combination of narration, visual demonstrations, graphics, animation, and written material. These features may make health information more accessible to individuals who have difficulty interpreting traditional written educational materials [8]. Videos can also be distributed through widely used platforms such as YouTube (Google LLC, Mountain View, California, United States) and social media or through physical formats such as DVDs, allowing health education to reach individuals in locations where in-person services may be limited.

Video-based interventions have been evaluated across multiple health topics and populations. Research has examined their use for cancer screening, infectious disease prevention, nutrition, physical activity, mental health literacy, and general health promotion [9-16]. Although not all studies were conducted specifically in rural US populations, the evidence provides insight into the potential mechanisms through which video-based education may improve health literacy and encourage preventive behaviors.

The purpose of this narrative review is to evaluate the evidence regarding health promotion videos and their effects on health literacy, health behaviors, and healthcare engagement, with particular attention to their potential application in rural and underserved populations in rural Alabama.

Contribution of this review

Existing reviews have examined video-based health education broadly or have focused on a single health behavior (e.g., cancer screening) or a single delivery platform (e.g., social media) [13,14,16-18]. This review’s contribution is threefold. First, it synthesizes evidence across health literacy, preventive screening, chronic disease self-management, and social media delivery to identify recurring design and implementation principles that apply across topics, rather than evaluating one behavior or platform in isolation. Second, it explicitly separates the small number of studies conducted in rural US populations from the larger body of broader supporting evidence so that conclusions about rural Alabama are appropriately hedged rather than inferred from populations that differ substantially from rural Alabama adults. Third, drawing on a comparison of two independent rural US studies, a cancer-screening trial and a veterans’ telehealth cohort study, it proposes a structural framework in which video-based education is paired with patient navigation or existing rural health infrastructure to address the access barriers that video content alone cannot resolve. This framework is intended to guide future implementation and evaluation efforts in rural Alabama, and its cross-study basis is limited enough that we present it as hypothesis-generating rather than confirmed.

## Review

Methods

A narrative review of the literature was conducted using PubMed and institutional academic databases as the primary sources of peer-reviewed evidence, supplemented by the Google search engine (Google LLC, Mountain View, California, United States) for a distinct and limited purpose described below. 

PubMed was used to identify peer-reviewed intervention studies, systematic reviews, meta-analyses, and qualitative studies examining the effects of digital or video-based health promotion media on health behaviors, health literacy, healthcare utilization, or health-related outcomes. Search terms included combinations of health promotion videos, rural populations, health literacy intervention, public health campaign, health education media, digital health promotion, social media education, and video-based health education. Searches were conducted between July 2025 and May 2026. 

Google was used only to identify epidemiologic, geographic, and policy context sources, for example, state health department reports, Centers for Disease Control and Prevention surveillance data, and geographic mapping resources describing medically underserved areas and community health center distribution in Alabama and the broader United States. Google was not used to identify the primary intervention studies synthesized in the Results, so that the effectiveness evidence in this review rests on peer-reviewed, PubMed-indexed sources. 

Screening was conducted in two stages. Titles and abstracts identified through the search terms above were first screened against the inclusion and exclusion criteria below; records that could not be confidently excluded at this stage underwent full-text review using the same criteria. 

Inclusion criteria were studies involving adolescents, adults, older adults, low-income populations, rural or underserved populations, or individuals with limited health literacy; interventions involving video or visual health education; instructional or health promotion content; and studies reporting qualitative or quantitative outcomes. Exclusion criteria were videos used solely for entertainment without a health education or public health purpose; interventions lacking a health literacy or health promotion component; articles not available in English; content consisting primarily of commercial advertisements without public health relevance; and studies not reporting measurable qualitative or quantitative outcomes. 

Because this is a narrative rather than a systematic review, a formal risk-of-bias instrument (e.g., Cochrane RoB 2, Newcastle-Ottawa Scale) was not applied. To support transparent interpretation of the evidence, study design (randomized controlled trial, systematic review or meta-analysis, qualitative study, or descriptive/epidemiologic source), approximate sample size, and peer-review status were recorded for each included source. Randomized controlled trials and systematic reviews were given greater interpretive weight than single-group, cross-sectional, or qualitative designs when synthesizing claims about effectiveness; this weighting is reflected in the hedged language used throughout the Results and Discussion, which distinguishes findings "demonstrated" in trials from patterns merely "suggested" or "associated" in lower-certainty designs. This approach does not substitute for the systematic quality appraisal used in a formal systematic review, and the conclusions of this narrative review should be weighed accordingly. 

Following application of these criteria, 20 sources were included in this narrative review: 13 peer-reviewed studies directly examining health education, health promotion, or digital health interventions, and seven additional sources providing epidemiologic, geographic, or contextual information on rural health and chronic disease in the United States and Alabama. Of the 13 intervention-focused sources, two were conducted in rural US populations (a randomized trial among rural cancer-screening-eligible women [12] and a retrospective cohort study among rural US veterans [19]); the remaining eleven were conducted in adolescent, college, university-personnel, or international populations and are treated in this review as supporting rather than direct evidence for rural Alabama. 

Results

Health Literacy and Knowledge

Mbanda et al.'s scoping review of 47 studies found that visual aids, including pictograms and videos, were associated with statistically significant improvements in health literacy outcomes among persons with low literacy, along with reported benefits for medication adherence and comprehension [8]. In a randomized controlled trial, Schröder et al. found that YouTube health videos improved mental health literacy among adolescents and teachers [9]. Kaneda et al.'s randomized controlled trial found that the simultaneous distribution of e-learning and exercise videos improved health literacy among college students during the COVID-19 pandemic [10]. Brame's synthesis of the instructional-video literature identified cognitive load management, engagement, and active learning as the three elements most associated with effective educational videos and cited an analysis of 6.9 million video-watching sessions in which median engagement fell from nearly 100% for videos under six minutes to approximately 50% for videos nine to 12 minutes and approximately 20% for videos of 12-40 minutes [11].

Preventive Health Behaviors

Champion et al.’s randomized clinical trial among rural women in Indiana and Ohio who were not up to date with breast, cervical, or colorectal cancer screening found that a mailed, tailored educational DVD increased screening completion at one year compared with usual care, and that adding telephonic patient navigation to the DVD produced a further increase in screening completion beyond the DVD alone [12]. Gujral et al.’s retrospective cohort study of rural US veterans found that those who received video-enabled tablets during the COVID-19 pandemic had more psychotherapy and video-based mental health visits and lower rates of emergency department visits and suicide-related outcomes than veterans who did not receive tablets [19]. Tuong et al.’s systematic review found evidence that video-based education can modify health behaviors across multiple health domains [13]. Boynito et al.’s systematic review and meta-analysis found that community-based educational video interventions were associated with effects on nutrition, health-related outcomes, and use of health services, primarily in low- and middle-income countries [14].

Lifestyle Behaviors and Chronic Disease Management

Udomkhwamsuk et al. evaluated an information-motivation-behavioral-skills video intervention among university personnel in Thailand during the COVID-19 pandemic and reported improvements in health-related outcomes [15]. Kaneda et al.’s trial, described above, also reported improvements in aspects of lifestyle alongside health literacy among college students [10]. No intervention study identified in this review was conducted specifically among rural Alabama adults; the state’s 13.9% adult-diagnosed diabetes prevalence and its documented medically underserved areas come from surveillance and geographic data sources rather than from a video-based intervention trial [4-7]. 

Social Media and Video-Based Health Promotion

Basch et al. found that TikTok (ByteDance Ltd., Los Angeles, United States) videos were used to disseminate handwashing information during the COVID-19 pandemic, demonstrating the reach of short-form video on that platform [16]. Zimmermann and Tomczyk’s qualitative interview and focus-group study found that adolescents use social media as an environment in which they encounter and engage with health information [17]. Koh et al.’s perspective piece characterized digital health promotion as carrying both promise and risk, raising concerns related to credibility, accessibility, privacy, and information quality [18].

Community-Based Interventions and Digital Access

Hearn et al.’s ACHIEVE (Advancing Community Health Innovation through Education, Vision, and Empowerment) program found that community engagement was associated with improved health literacy among youth in rural Pennsylvania, although the intervention was not video-specific [20]. Champion et al.’s trial delivered its DVD intervention by mail rather than online, reaching rural women regardless of home broadband access [12].

Discussion

The findings of this narrative review suggest that health promotion videos represent a promising complementary strategy for improving health literacy, preventive behaviors, and engagement with health information among rural and underserved populations. Evidence is strongest for improvements in knowledge and health literacy, while evidence for sustained behavioral and clinical outcomes is more variable and, for rural US populations specifically, rests on only two identified intervention studies. This section interprets those findings and considers their implications for rural Alabama; it does not restate the study-by-study findings reported in Results.

Health Literacy as an Intermediate, Not Terminal, Outcome

Health literacy is a plausible mechanism through which video interventions could influence health behaviors, since individuals who better understand disease risk, preventive recommendations, and treatment options may be better equipped to make informed decisions. However, the reviewed evidence indicates that health literacy gains do not necessarily translate into sustained behavior change. Brame’s engagement-time data are informative here: if attention drops sharply after six minutes, even well-designed content has a narrow window in which to convey actionable information, which constrains what any single video can be expected to achieve on its own [11]. Knowledge should therefore be treated as an intermediate outcome, with actual behavior and clinical outcomes shaped by a wider set of social, economic, environmental, and healthcare-access factors that video content cannot address directly. 

From Information to Action: A Hypothesis-Generating Pattern Across Two Rural US Trials

The Champion et al. and Gujral et al. studies are, to our knowledge, the only two intervention studies identified in this review conducted in rural US populations, and both compared conditions that isolate the value of pairing video-based or video-enabled technology with a mechanism for accessing care [12,19]. In Champion et al., DVD education alone increased screening relative to usual care, but adding patient navigation and direct assistance to overcome scheduling and access barriers produced a further increase beyond the DVD alone [12]. In Gujral et al., the video-enabled tablets did not deliver health content directly; instead, they functioned as an access mechanism connecting rural veterans to existing telehealth mental health services, and this access was associated with more service use and fewer adverse outcomes [19]. Taken together, these two trials suggest a pattern in which the observed benefit is larger when video or video-enabled technology is paired with something that resolves a structural access barrier, navigation in one case and telehealth connectivity in the other, rather than education or technology access alone. With only two trials, in different populations and different health domains, this pattern should be treated as hypothesis-generating rather than established, and it has not been tested directly in rural Alabama. 

Designing Effective Rural Health Promotion Video Content

Applying Brame’s principles of managing cognitive load, sustaining engagement, and supporting active learning [11] alongside Champion et al.’s use of content tailored to participants’ specific risks, barriers, and beliefs [12] suggests that rural health promotion videos should be concise, focused on a single actionable behavior, and tailored to locally relevant barriers rather than presenting general medical information. Community engagement in development, as described by Hearn et al. [20], may further improve relevance and trust and could help identify the specific barriers (transportation, cost, scheduling) that a companion navigation or referral component would need to address. 

Social Media: Reach Versus Credibility

The reach demonstrated by Basch et al. and the everyday use described by Zimmermann and Tomczyk indicate that social media could extend health messaging to rural residents, including younger populations, without requiring a clinical encounter [16,17]. However, Koh et al.'s concerns about credibility, accessibility, privacy, and information quality apply directly to this delivery channel [18]: a social-media strategy for rural Alabama would need explicit sourcing, review, and messaging safeguards to avoid competing with, rather than supplementing, accurate health information.

Applying the Evidence to Rural Alabama: A Proposed Framework

No study identified in this review directly tested video-based health promotion among rural Alabama adults. What follows is a proposed application of the broader evidence base, not a demonstrated Alabama-specific effect. Alabama’s 13.9% adult-diagnosed diabetes prevalence and documented medically underserved areas [4-7] describe a population with both elevated chronic disease burden and constrained access to in-person care, conditions similar in kind, though not identical, to those in the Champion et al. and Gujral et al. study populations. Consistent with the pattern described above, a video-based program in rural Alabama would plausibly benefit from pairing concise, tailored video content (for diabetes, cardiovascular risk, cancer screening, or tobacco cessation) with an explicit access component, community health center-based navigation, scheduling assistance, or telehealth connectivity, rather than distributing video content alone. This framework should be tested directly, ideally with comparative design similar to Champion et al.’s DVD-only versus DVD-plus-navigation arms, before it is adopted as a program model. 

Scalability and Cost

Champion et al.’s trial demonstrates that a remotely delivered video intervention can be implemented at a population scale and evaluated for cost-effectiveness [12], which is relevant to rural areas facing healthcare workforce shortages, since standardized video content could supplement, rather than replace, staff-delivered education. This cost-effectiveness evidence, however, comes from a single US trial in one population and health domain; whether comparable cost-effectiveness would hold for a diabetes- or cardiovascular-focused rural Alabama program has not been tested and should not be assumed. 

Limitations

Several limitations should be considered when interpreting this narrative review. First, only two of the 13 intervention-focused sources were conducted in rural US populations [12,19]; the remaining eleven were conducted in adolescent, college, university-personnel, or international populations, which limits direct generalization to rural Alabama adults despite their value in establishing mechanism and feasibility [9-11,13-18]. Second, several interventions measured knowledge, attitudes, intentions, or self-reported behaviors rather than objective clinical outcomes, and improvements in health literacy may not translate into sustained changes in disease outcomes. Third, some interventions incorporated additional components, such as patient navigation, making it difficult to isolate the contribution of the video component itself [12]. Fourth, as a narrative rather than a systematic review, no formal risk-of-bias instrument was applied, and the search, while conducted across PubMed and supplementary sources, was not exhaustive; a formal systematic review with structured quality appraisal would provide stronger evidence than is possible here. Fifth, digital access itself presents a limitation, as inadequate broadband, limited device availability, and lower digital literacy may restrict the reach of online interventions. Finally, because only two rural US intervention studies were identified, and only one addressed a chronic-disease-relevant behavior context comparable to Alabama’s, the structural framework proposed in the Discussion is hypothesis-generating and has not been tested in rural Alabama specifically. 

Future directions

Future research should prioritize studies conducted specifically in rural Alabama and comparable rural US communities, ideally using comparative designs similar to Champion et al.’s DVD-only versus DVD-plus-navigation arms, to directly test whether pairing video content with an access-oriented component (navigation, telehealth, connectivity, or community health center referral) produces larger effects than video content alone. Longitudinal designs are needed to determine whether short-term improvements in knowledge and health literacy persist and translate into sustained behavior change and objective outcomes such as screening completion, vaccination rates, documented healthcare utilization, and medication adherence, rather than relying solely on self-reported measures. A future systematic review with formal quality appraisal (e.g., Cochrane RoB 2 or a comparable instrument) would also strengthen confidence in these findings beyond what a narrative review can provide. Partnerships between academic institutions, local health departments, community health centers, schools, and community organizations could facilitate the development and evaluation of locally relevant interventions, with resident and clinician involvement helping to identify priority health concerns, barriers to care, and appropriate delivery platforms. Finally, research should continue to examine the credibility and quality of health information distributed through social media, as platforms such as YouTube and TikTok become more prominent sources of health information for rural and younger populations alike. 

## Conclusions

Health promotion videos represent a promising, scalable, and potentially cost-efficient strategy for improving health literacy and supporting preventive health behaviors among rural and underserved populations. This review’s contribution is not a new primary study or a general restatement of known video-education benefits; it is a synthesis of evidence across health literacy, preventive screening, chronic disease self-management, and social media delivery that explicitly separates the small body of rural US intervention evidence from broader supporting literature and a resulting structural framework, pairing video-based education with navigation or existing rural health infrastructure, proposed for rural Alabama on the basis of a pattern observed across two rural US trials. Video-based education cannot independently resolve transportation barriers, healthcare workforce shortages, financial constraints, or inadequate broadband access. Future research conducted directly in rural Alabama, using comparative designs and outcome measures, is needed to determine whether the proposed framework produces the sustained improvements in health behavior and population health that this review hypothesizes. 
